# Waiting Times for Prostate Cancer Diagnosis in a Nigerian Population

**DOI:** 10.1155/2021/5534683

**Published:** 2021-08-16

**Authors:** Olufunmilade A. Omisanjo, Olawale O. Ogunremi, Olufemi O. Akinola, Olaolu O. Adebayo, Olufemi Ojewuyi, Mofeyisayo O. Omorinde, Abimbola A. Abolarinwa, Stephen O. Ikuerowo, Fatai A. Balogun

**Affiliations:** Department of Surgery, Lagos State University College of Medicine and Teaching Hospital (LASUCOM/LASUTH), Ikeja, Lagos, Nigeria

## Abstract

**Background:**

Prostate biopsy remains an important surgical procedure in the diagnostic pathway for prostate cancer, but access to prostate biopsy service is poorly studied in the Nigerian population. While there has been a well-documented delay in patient presentation with prostate cancer in Nigeria, little is however known about how long patients wait to have a histological diagnosis of prostate cancer and start treatment after presenting at Nigerian hospitals.

**Method:**

This was a descriptive retrospective study to document the specific duration of the various timelines in getting a diagnosis of prostate cancer at the Lagos State University Teaching Hospital, Ikeja, Nigeria.

**Results:**

There were 270 patients. The mean age was 69.50 ± 8.03 years (range 45-90). The mean PSA at presentation was 563.2 ± 1879.2 ng/ml (range 2.05-15400), and the median PSA was 49.3 ng/ml. The median waiting times were (i) 10 days from referral to presentation; (ii) 30 days from presentation to biopsy; (iii) 24 days from biopsy to review of histology; (iv) 1 day from histology review to discussion/planning of treatment. The median overall waiting time from referral to treatment was 103 days. The mean time from presentation to biopsy was significantly shorter for patients with PSA of ≥50 ng/ml compared to those with PSA < 50 ng/ml. *p* = 0.048. Overall, the median time from biopsy to histology was significantly shorter for patients whose specimens were processed in private laboratories (17 days) compared to those whose specimens were processed at the teaching hospital laboratory (30 days), *p* ≤ 0.001.

**Conclusion:**

There is a significant delay within the health care system in getting a prostate cancer diagnosis in the Nigerian population studied. The major points of the identified delay were the waiting time from patient presentation to having a biopsy done and the histology report waiting time.

## 1. Background

Prostate cancer is documented as the second most frequently diagnosed cancer in men globally [1]. Amongst Nigerian males, it is the most commonly diagnosed cancer, accounting for 11% of all male cancers [2]. Prostate biopsy remains the gold standard for the diagnosis of prostate cancer despite recent improvements in imaging such as multiparametric magnetic resonance imaging. After patient referral and initial evaluation, a prostate biopsy is usually scheduled for histological confirmation of the cancer, and appropriate treatment is then planned and subsequently implemented. Sometimes delays are encountered during the diagnostic process, treatment planning, and implementation.

The waiting time for establishing a diagnosis of prostate cancer and instituting treatment varies with country and sometimes between different regions in the same country [3–6]. Generally, there is often public concern about cancers and how quick and easy it is for patients with cancer to assess care [7]. In addition, a patient's perceived waiting time for cancer treatment has been shown to be important for patient satisfaction [8]. A number of reasons have been adduced as being responsible for these various delays in getting a prostate cancer diagnosis and treatment.

Much has been written about the late presentation of patients with prostate cancer in Nigeria—either patients presenting with the disease ab initio or those presenting with castrate resistance [9–11]. Little is however known about how long patients have to wait to confirm a diagnosis of prostate cancer and get treatment commenced after presenting at hospitals in our environment. While there are several complex patient-related factors for delays in seeking cancer diagnosis, there are also health system-related factors which may contribute to delays in establishing cancer diagnosis and treatment. Recently, in a study involving patients with breast and cervical cancers in Lagos, Nigeria, Awofeso et al. showed that while poor health seeking patient behavior was contributory to late disease presentation and diagnosis, there were also significant health system delays [12]. Identifying the possible points of delay within the health care system that hinder a rapid establishment of a prostate cancer diagnosis is vital to promulgating policies that may help reduce these delays and improve service efficiency. In addition, having adequate waiting statistics helps to monitor performance against waiting time targets [13].

There is no study amongst the Nigerian population detailing the specific timelines for getting a diagnosis of prostate cancer. This study is aimed therefore at documenting the specific duration of the various timelines in getting a diagnosis of prostate cancer at the Lagos State University Teaching Hospital, Ikeja, Nigeria.

## 2. Patients and Methods

The study was a 5-year retrospective one spanning January 2013 to December 2017. The clinical records of all patients who had prostate biopsy within the study period at the Lagos State University Teaching Hospital, Ikeja, were retrieved from their medical case files. Data analyzed included patients' age, PSA, histology, and type of laboratory where the specimens were processed. The timelines we studied were the duration of (i) from time of patient referral to the time of patient presentation at the teaching hospital; (ii) time from patient presentation to having a prostate biopsy done; (iii) time from having prostate biopsy done to receiving the histology report; and (iv) time from receiving a histology report to discussing/planning treatment with the urologist.

The data were captured with Microsoft Excel, and data analysis was performed by Statistical Package for Social Sciences (SPSS) version 20.0 for Windows. The data were expressed as means and medians with the test for statistical significance carried out by Fischer's exact test, chi-square test, and the median test. A *p* value < 0.05 was considered significant.

## 3. Results

**Figure 1:**
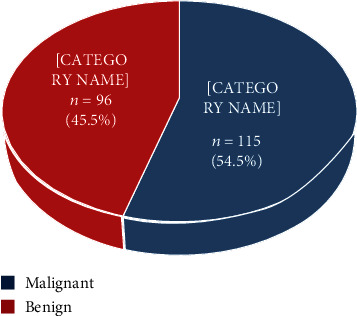
Pie chart showing histology report of prostate biopsies.

Records were available for 270 patients. The mean age was 69.50 ± 8.03 years (range 45-90). The mean PSA at presentation was 563.2 ± 1879.2 ng/ml (range 2.05-15400), and the median PSA was 49.3 ng/ml. Of the 211 definitive histology reports available, the histology was malignant for 115 patients (54.5%) (Figure 1).

The median PSA of the patients diagnosed with prostate cancer (140 ng/ml) was significantly higher than that of the patients diagnosed as benign prostatic hyperplasia (18.5 ng/ml), *p* ≤ 0.001.

The median waiting times were (i) 10 days from referral to presentation; (ii) 30 days from presentation to prostate biopsy; (iii) 24 days from biopsy to review of histology; (iv) 1 day from histology review to discussion/planning of treatment (Table 1).

The median overall waiting time from referral to discussing/planning treatment was 103 days (93 days for patients with a diagnosis of carcinoma of the prostate and 107 days for patients with a diagnosis of benign prostatic hyperplasia, *p* = 0.372) (Tables 1 and 2).

The patients with a PSA of ≥50 ng/ml had a significantly shorter period to have their biopsy done after presentation (mean: 25.5 days) compared to those patients with PSA < 50 ng/ml (mean: 32.2 days), *p* = 0.048.

Overall, the median time from biopsy to histology was significantly shorter for patients who had their specimens processed in private laboratories (17 days) compared with those who had their specimens processed at the teaching hospital laboratory (30 days), *p* ≤ 0.001 (Table 3).

## 4. Discussion

Prostate biopsy remains an important surgical procedure in the diagnostic pathway for prostate cancer, but access to prostate biopsy service is poorly studied in the Nigeria population. Our study documents clearly the various timelines for getting a diagnosis of prostate cancer established in a Nigerian population. The patient population in our study was similar to those of previous reports on prostate cancer in Nigeria. The mean age of 69.5 years that we found was similar to the 71.4 years and 68 years found by Ogunbiyi and Shittu and Badmus et al., respectively [2, 9]. The relatively high PSA amongst the study population is also in keeping with the previously documented late presentation of prostate cancer in our environment [9, 10].

The waiting time for establishing a prostate cancer diagnosis (median of 93 days or 13.3 weeks) in our studied population is amongst the longest documented in the literature so far. The waiting time we found is much longer than the documented figures of 7.7 weeks and 7.6 weeks found in Poland and Canada, respectively [4, 14]. Our waiting time is however similar to the waiting time of 100 days found by Singh et al. in a South African population [5]. The waiting time for cancer diagnosis is considered by some to be a measure of the quality of health care delivery. Waiting times are generally taken as a good indicator of the quality of cancer care [3]. This long waiting time in our study population is probably a reflection of the poorly organized health service delivery system in our country with generally poor health indices not necessarily limited to oncological care. Similar delays have been documented for diagnosing breast and cervical cancers in Nigeria [12, 15].

The impact of a long waiting time for prostate cancer diagnosis on the outcome of treatment of the disease has been conflicting. While Abern et al., Nguyen et al., and O'Brien et al. all documented some form of a negative impact on treatment with treatment delay [16–18], other workers found no significant impact of treatment delay on oncologic outcomes [19–21]. It is however important to note that these findings were majorly in studies involving patients with early disease. In our environment with well-documented late presentation with prostate cancer, it is reasonable to expect that any further delay in confirming the diagnosis and initiating treatment can only make the outcome worse, possibly in terms of quality of life issues, if not in terms of disease specific mortality.

The impact of a long waiting time on the psychology of patients with prostate cancer is however much clearer. Prostate cancer diagnosis has been documented to be associated with anxiety and emotional distress [22–24]. Identifying the main points of delay in prostate cancer diagnosis and putting in place measures to shorten such periods can potentially reduce patients' distress. When we compared the various timelines for the patients, the time from presentation to biopsy and the time spent awaiting the histology report were of the longest duration. These delays come with possible negative implications. The prebiopsy waiting time has been documented to have an impact on patients' anxiety levels and pain perception when a prostate biopsy is eventually carried out. Saracoglu et al. found a prebiopsy waiting of more than 10 days to be significantly associated with a higher anxiety level and pain perception during prostate biopsy compared with patients with a shorter waiting time [25].

One of the other findings of our study was that patients with PSA ≥ 50 had a significantly shorter time between presentation and getting their prostate biopsy done. The plausible reason for this is that these patients with very high PSAs (sometimes in the thousands as shown in the range of PSA) present to us very sick and end up being admitted into our inpatient service for resuscitation and sometimes treatment of complications such as cord compression and haematuria. Admission into our inpatient service offers such patients a faster route to having a prostate biopsy done compared to those patients with lower PSA who generally remain as outpatients.

The waiting time for histology reporting that we found was also long. Decreasing the amount of time needed to get the histology report is also vital to reducing the anxiety associated with a prostate cancer diagnosis. Dale et al. showed in a review that when anxiety in prostate cancer is studied over clinical timelines, patients generally have the highest documented level of anxiety while awaiting the histology results—with some reduction in anxiety levels once the histology report is available whether it is positive or negative for cancer [26]. This reduction in anxiety after the receipt of the histology report is thought to be due to some anxiolytic effect of information receipt to eliminate uncertainty irrespective of whether the information received is the preferred outcome or not [27, 28].

Over the years, different strategies have been documented to reduce waiting times for cancer diagnosis. In the UK, the NHS Cancer Plan and the Cancer Reform Strategy were programmes formulated, respectively, in 2000 and 2007 to expedite cancer diagnosis, improve cancer survival, and decrease attendant patient anxiety [29, 30]. A Canadian study also showed that a Calgary rapid access clinic reduced the waiting time between referral and biopsy by 78% in patients with prostate cancer [31]. In 2015, Norway introduced a dedicated cancer patient pathway in an attempt to reduce waiting times for treatment [32]. Any programme that is aimed at reducing waiting time for cancer diagnosis has to focus on potentially easily modifiable timelines in the diagnostic pathways in that population. Another major health system point of delay that our study identified was the histology reporting time. We found the time for reporting of histology to be significantly shorter in patients who had their prostate biopsy specimens processed in certified private laboratories compared with those who had their specimens processed in our public hospital. This finding offers a potential target for reducing the waiting time. A number of government run hospitals in Nigeria now have a public-private partnership framework that allows for some private sector involvement in public hospital health service delivery. We advocate that strong consideration has to be given to possibly allowing more private sector participation in specimen handling in Nigerian public hospitals and thereby shortening the time for processing pathology specimens and getting a histology report.

Our study is limited in being a retrospective review in a single institution. Our paper has however highlighted some of the health system delays that have hitherto been poorly documented in our region. Our study has clearly documented the relative delay in establishing prostate cancer diagnosis in our patient population when compared to previous documentation in other regions. We have also identified potentially modifiable timelines that may help with mitigating against these delays. The delay we have identified within the health care system strengthens the argument for some form of a national or regional cancer plan dedicated to reducing the waiting times for prostate cancer diagnosis and treatment in Nigeria. Such programmes have been successfully carried out in other countries [29–32].

## 5. Conclusion

There is a significant delay in getting a prostate cancer diagnosis in the Nigerian population studied. These delays within the health care system are potentially modifiable. There is a need for a comprehensive review of the oncology care delivery system with great attention paid to reducing the health system-related delays identified with the timelines in getting a diagnosis of prostate cancer, particularly the waiting time from patient presentation to having a biopsy done and the histology report waiting time.

## Figures and Tables

**Table 1:** Waiting times for all patients.

*S*/*N*	Parameter	Mean ± SD	Range	Median (IQR)
1	Referral–presentation (days)	28.09 ± 44.74	1–263	10.00 (27.00)
2	Presentation–biopsy (days)	56.98 ± 73.79	1–510	30.00 (62.00)
3	Biopsy–histology (days)	37.30 ± 39.75	4–330	24.00 (26.00)
4	Histology–treatment (days)	13.08 ± 32.02	0–300	1.00 (14.00)
5	Presentation–treatment (days)	107.36 ± 98.03	9–840	83.00 (89.00)
6	Referral–treatment (days)	135.45 ± 118.77	10–934	103.00 (126.00)

SD: standard deviation.

**Table 2:** Waiting times based on patient histology.

*S*/*N*	Parameter			Benign *n* = 96	Malignant *n* = 115	*p* value
1	Age (years)		Mean ± SD	67.71 ± 7.52	71 ± 8.17	0.003
			Median (IQR)	68.00 (8.75)	71.00 (13.00)	0.043
2	PSA (ng/ml)		Mean ± SD	49.22 ± 157.57	992.30 ± 2465.02	0.000
			Median (IQR)	18.50 (24.70)	140.00 (693.00)	0.000
3	Waiting time (in days)	Referral–presentation	Mean ± SD	29.58 ± 51.23	26.84 ± 38.69	0.667
			Median (IQR)	8.00 (24.75)	13.00 (27.00)	0.105
4		Presentation–biopsy	Mean ± SD	82.21 ± 93.70	35.92 ± 41.60	0.000
			Median (IQR)	53.00 (103.25)	21.00 (48.00)	0.000
5		Biopsy–histology	Mean ± SD	39.79 ± 45.17	35.23 ± 34.65	0.418
			Median (IQR)	24.00 (28.00)	24.00 (21.00)	0.554
6		Histology–treatment	Mean ± SD	0.28 ± 1.94	23.77 ± 40.41	0.000
			Median (IQR)	0.00 (0.00)	14.00 (24.00)	0.000
7		Presentation–treatment	Mean ± SD	122.28 ± 116.27	94.91 ± 78.06	0.043
			Median (IQR)	89.00 (126.00)	77.00 (74.00)	0.242
8		Referral–treatment	Mean ± SD	151.86 ± 141.29	121.76 ± 94.52	0.067
			Median (IQR)	107.00 (139.25)	93.00 (102.00)	0.372

SD: standard deviation; IQR: interquartile range.

**Table 3:** Crosstabulation of various timelines with the type of laboratory where histology was done.

		Private lab *n* = 80	Public lab *n* = 131	*p* value
		Frequency (%)	Frequency (%)	
Biopsy to histology	<8 days	6 (7.5)	0 (0.0)	0.000^∗^
	8–14 days	25 (31.3)	9 (6.9)	
	>14 days	49 (61.3)	122 (93.1)	
	Mean ± SD	28.49 ± 33.28	42.69 ± 42.45	0.007
	Median	17.00 (18.00)	30.00 (28.00)	0.000

Presentation to treatment	<28 days	17 (21.3)	8 (6.1)	0.000
	28–90 days	39 (48.8)	54 (41.2)	
	>90days	24 (30.0)	69 (52.7)	
	Mean ± SD	84.65 ± 83.36	121.24 ± 103.87	0.005
	Median	55.00 (71.00)	91.00 (93.00)	0.000

Referred to treatment	<28 days	13 (16.3)	5 (3.8)	0.000
	28–90 days	36 (45.0)	41 (31.3)	
	>90 days	31 (38.8)	85 (64.9)	
	Mean ± SD	105.98 ± 107.27	153.46 ± 122.21	0.004
	Median	71.50 (96.25)	119.00 (121.00)	0.001

## Data Availability

The datasets used and/or analyzed during the current study are available from the corresponding author on reasonable request.
